# Report of the Diseases of Edinburgh for March, 1808

**Published:** 1808-05

**Authors:** John Roberton

**Affiliations:** Surgeon


*Report of the Diseases of Edinburgh for March,* 1808. By JOHN ROBERTSON, Surgeon.

This month commenced with very mild and pleasant weather. In a few days, however, cold east winds, with thick fogs, particularly during the night, prevailed. About the end of the first third of the month, the weather became very cloudy, and it still continued cold. Snow appeared on the neighbouring hills, and we had some smart gales of wind wind from about east, which rendered the weather very unpleasant as well as very cold. The changes of temperature which took place, were indeed very few during the whole month, and the easterly wind for the most part prevailed. Toward the termination of the month we had a considerable fall of snow, which was likewise pretty general over this part of the country, and put a stop for some days to the labors of the farmer.

The thermometer, during the first half of the month, stood high, independent of the several changes in the state of the weather. It began to fall about the beginning of the last third, and rose again toward the end; and when the month terminated, it was about fair.

Soon after the commencement of the month, the measles, which has lately made such a conspicuous figure in our catalogue of diseases, seemed to have nearly exhausted itself, as I believe very few persons escaped who had been previously unaffected by this disease. The cases, however, which did occur, were of a very bad kind, and many of the patients soon after died of pulmonic complaints. This did not often happen among children, but among people somewhat advanced in life.

About the beginning of the month, the Inverness-shire Militia marched into this place, and, as will appear from the following statement given me by my friend Mr. John Campbell, many of them suffered severely from it.

" Edinburgh, April, 1808.

" The disease, [the measles,] was epidemic in town when the regiment arrived: upwards of fifty men were in the course of a few nights seized with it; and it is remarkable, that all these were recruits just joined; the other men escaping the disease, although equally exposed to it.

" The disease was unusually formidable, owing, I believe, rather to the subjects whom it attacked, being young plethoric men newly arrived from the country, than to any peculiarity in the disease itself; for the form under which it generally appeared, was that of the Rubeola Vulgaris, one case only of the Rubeola sine Catarrho having occurred; nor have I ever met with the measles described by Sir W. Watson in the London Medical Observations.

" From the unusually great determination to the lungs, which took place in most of the cases under my observation, and the danger to be dreaded from the supervention of pulmonic inflammation, blood-letting seemed to be strongly indicated, and accordingly it was had recourse to, with evident advantage; in some cases the lancet was used, in others cupping. It appeared that this remedy was most beneficial when employed according to the urgency of the symptoms, as recommended by Heberden and Mead; and not when reserved for a particular period of the disease, as advised by Sydenham and Morton. It is a remedy which undoubtedly ought not to be indiscriminately had recourse to in this disease, and seems justified only upon the supervention of dyspnœa and other symptoms of pneumonic inflammation.

"The other remedies from which most benefit was derived, were blisters, laxatives, mild tepid drinks, together with a light diet, and bathing the feet every night in warm water. The inspiration of steam seemed to alleviate the Catarrhal symptoms more than any of the remedies I have seen usually prescribed for that purpose.

"Upon opening the thorax of some of those who died labouring under pneumonic affections, I found the following appearances.

"The plura costalis, plura pulmonalis, and pericardium very much inflamed; frequent and extensive adhesions between the pluræ. An exudation of viscid whitish fluid covering every where the surface of the pluræ; a similar exudation had taken place into the bronchiæ; a preternatural effusion of serous fluid appeared likewise to have taken place into the pericardium."

Pulmonary complaints, attended by a very considerable degree of fever, have been very common, even with those who were not previously affected with measles. Owing to the great debility with which the patients were affected, even from the commencement of this disease, we were seldom, I believe, warranted to employ general blood-letting. In one or two cases, indeed, I found it absolutely necessary, but the debility which ensued in these cases, ought to caution us against such practice, unless in such cases as I have alluded to, where, almost certainly, the existence of the patient must have terminated in a very few hours, if such active measures had not been adopted.

It is in such cases, that the medical attendant shews himself, in the line of his profession, superior, or otherwise. It is then that his discernment and his description must show him to be unswayed by the hackneyed rules which have too long disgraced our profession, and when other arts and sciences are rapidly emerging from darkness and doubt, effectually prevent its improvement.

Toward the end of the month, scarcely any cases of measles were to be met with. I may

I may here remark, that the late frequency of the measles in this place has been *most satisfactorily* accounted for. It has been said entirely to depend on the *ridiculous* and *unsafe* practice of inoculating with cow-pox; and I am sorry to say, that this opinion has not been entirely confined to those who make up for their want of reason and discernment by their multitude. The question, whether or not this really was likely to be the fact, has been put to me by those whose situation in society, and acquirements in other matters, entitled us to expect opinions from them of a more liberal nature. This may serve as an additional instance how easily the *multitude* may be swayed; for at this moment, so convinced are the people, about this place, of the above statement being true, that, for several weeks past, the inoculation for the cow-pox has proceeded very slowly, and consequently matter for that purpose is scarcely to be obtained.

The Catarrh, mentioned in former Reports, seemed to increase in frequency as well as in severity, and the great prostration of strength which followed it, was very remarkable. For the first day or so, the strongest inflammatory symptoms prevailed, which often carried off the patients in a very few days, and, if their lives were protracted beyond the first week or ten days by blood-letting, blistering, purgatives, or other means, a very great proportion of them were seized by dropsy in every variety of form. Some of those patients who have been affected by this Catarrh, in a very severe degree, have, on the abatement of the symptoms, been affected with all the different forms of dropsy at once, which I have rarely succeeded in completely removing, owing to the depraved state of action into which all the functions of the body had been thrown.

A sort of Ophthalmia has prevailed, particularly in the Edinburghshire Militia, stationed in this neighbourhood, which, by many, has been thought to resemble, in several particulars, the Egyptian Ophthalmia. It first originated at Dunbar, where the regiment was then stationed, but became much worse on their arrival in this neighbourhood, and was evidently epidemic. It has been said, that washing the eyes of those who had been exposed to the spray of the sea, or marching on sandy soil, had a very considerable effect in preventing, or moderating it. But when, in this case, the disease had fairly established itself, the patients got well only by one uniform mode of treatment. The eyes, both of which, in almost every instance, were severely affected, were freely scarified, so that no enlarged blood-vessel was left running over the surface of the eye-ball. This was was followed by the application of solutions of acet. plumbi and smart purgatives. In a few instances, indeed, it was found necessary, when this did not seem to complete the cure, to put a few drops of tinct. opii into the eye, or to apply ointment containing red precipitate of mercury, which, at all times, completed the cure.

At every period of the year, stomach complaints are here very common; but I have always observed, that from one to three or four months after the commencement of the year, when, according to the absurd custom of this part of the world, drinking in particular is a species of irregularity too common among us, these complaints are always more frequent and more obstinate than at any other time. Indeed, the immoderate use of ardent spirits in this country is much too common, and I will venture to say, that this is productive not only of stomach complaints, but of, perhaps, the greater proportion of the diseases that come under the care of the physician. Although Dyspepsia, in its most distressing and obstinate form, is almost always the first effect of this practice, it is soon followed, if the habit is persevered in, by an almost complete derangement of the digestive organs, accompanied by great torpidity of the bowels. The body, from this derangement, being deprived of its wonted nourishment, other diseases soon follow, and the wretched and almost unpitied sufferer lingers out a few years, equally insensible to every kind of happiness but the prospect of again reducing himself to a state of insensibility by intoxication.

Those unaccustomed to live in a country, and among people who use such immense quantities of ardent spirits as we are in the habit of using, are apt to imagine, that, after the continued use of this destructive potion, they are at last destroyed by the almost immediate effects of one of their doses; but this is not the case, for with us, where I am sure there is more ardent spirits consumed than in any other part of the world, it will be found that an amazing number of lingering, yet fatal, diseases are produced by the use of it, and where one person suffers immediate death by this means, many thousands drag out a miserable existence, unable either to continue their former practices, or to recover from the effects produced on the withered and emaciated fabric. The opinion that ardent spirits terminate the existence of the individual suddenly will be found to be formed not according to fact, but according to the opportunities the practitioner has, of, perhaps, seeing a few solitary instances of sudden death from apoplexy in consequence of drinking.

